# Dynamic Contrast-Enhanced Study in the mpMRI of the Prostate—Unnecessary or Underutilised? A Narrative Review

**DOI:** 10.3390/diagnostics13223488

**Published:** 2023-11-20

**Authors:** Silva Guljaš, Zdravka Dupan Krivdić, Maja Drežnjak Madunić, Mirela Šambić Penc, Oliver Pavlović, Vinko Krajina, Deni Pavoković, Petra Šmit Takač, Marin Štefančić, Tamer Salha

**Affiliations:** 1 Clinical Department of Radiology, University Hospital Centre, 31000 Osijek, Croatia; silva.guljas@gmail.com (S.G.); zdravka.krivdic@gmail.com (Z.D.K.); 2 Faculty of Medicine, Josip Juraj Strossmayer University of Osijek, 31000 Osijek, Croatia; majadreznjakmadunic@gmail.com (M.D.M.); mirela.penc@gmail.com (M.Š.P.); oliver.pavlovic@kbco.hr (O.P.); krajina.vinko@gmail.com (V.K.); deni.pavokovic@gmail.com (D.P.); 3 Department of Oncology, University Hospital Centre, 31000 Osijek, Croatia; 4 Department of Urology, University Hospital Centre, 31000 Osijek, Croatia; 5 Clinical Department of Surgery, Osijek University Hospital Centre, 31000 Osijek, Croatia; psmit292@gmail.com; 6 Department of Radiology, National Memorial Hospital Vukovar, 32000 Vukovar, Croatia; stefancic.marin@gmail.com; 7 Department of Teleradiology and Artificial Intelligence, Health Centre Osijek-Baranja County, 31000 Osijek, Croatia; 8 Faculty of Dental Medicine and Health, Josip Juraj Strossmayer University of Osijek, 31000 Osijek, Croatia

**Keywords:** magnetic resonance imaging, DCE-MRI, bpMRI, mpMRI, prostate cancer

## Abstract

The aim of this review is to summarise recent scientific literature regarding the clinical use of DCE-MRI as a component of multiparametric resonance imaging of the prostate. This review presents the principles of DCE-MRI acquisition and analysis, the current role of DCE-MRI in clinical practice with special regard to its role in presently available categorisation systems, and an overview of the advantages and disadvantages of DCE-MRI described in the current literature. DCE-MRI is an important functional sequence that requires intravenous administration of a gadolinium-based contrast agent and gives information regarding the vascularity and capillary permeability of the lesion. Although numerous studies have confirmed that DCE-MRI has great potential in the diagnosis and monitoring of prostate cancer, its role is still inadequate in the PI-RADS categorisation. Moreover, there have been numerous scientific discussions about abandoning the intravenous application of gadolinium-based contrast as a routine part of MRI examination of the prostate. In this review, we summarised the recent literature on the advantages and disadvantages of DCE-MRI, focusing on an overview of currently available data on bpMRI and mpMRI, as well as on studies providing information on the potential better usability of DCE-MRI in improving the sensitivity and specificity of mpMRI examinations of the prostate.

## 1. Introduction

**Figure 1:**
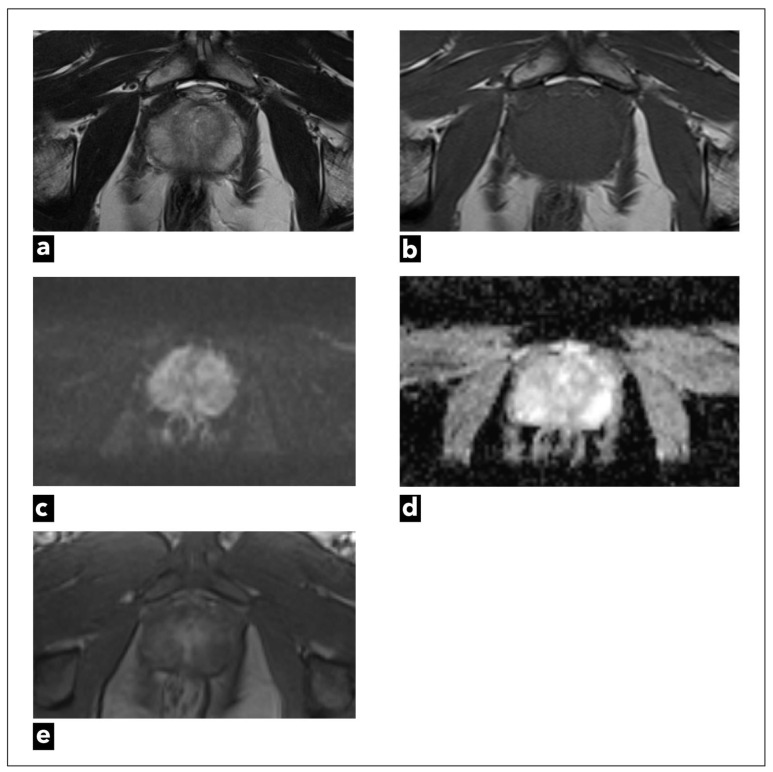
A 47-year-old male patient underwent mpMRI due to a slightly elevated serum PSA level. There were no suspicious findings. Figures present original examples of standard anatomical and functional sequences, respectively: (**a**) T2W and (**b**) T1W; (**c**) DWI; (**d**) ADC map; and (**e**) DCE.

Multi-parametric magnetic resonance imaging (mpMRI) is a widely used detection method for diagnosing clinically significant prostate cancer [1]. It has been proven to be very useful in localizing prostate cancer, navigating prostate biopsy, and staging prostate cancer. The examination includes anatomical T1 and T2 weighted (T1W, T2W) sequences and functional sequences—diffusion-weighted imaging (DWI), dynamic contrast-enhanced MRI (DCE-MRI), and magnetic resonance spectroscopic imaging (MRSI) [2] (Figure 1). All sequences, in combination, help navigate the final diagnosis. Anatomical sequences provide information on the morphology of the prostate and the surrounding tissue. In addition, mpMRI provides functional information on the metabolic activity of prostate cancer using spectroscopy, information on tumour angiogenesis using DCE-MRI, and information on cancer cellularity using the diffusion technique. 

DWI and its apparent diffusion coefficient map (ADC) are currently the main functional sequences that give us information on cancer cellularity [3]. The advantages of DWI and the corresponding ADC map are short acquisition times and high contrast resolution, which distinguishes potentially malignant tissue from benign tissue [4]. Some studies have even shown an inverse correlation between Gleason score and ADC values, which gives information on tumour aggressiveness [5,6]. Studies that have investigated quantitative ADC metrics, mainly focusing on the peripheral zone, have concluded that values above 900 mm^2^/s are likely benign, while values below 750 mm^2^/s are probably malignant [7].

In the Prostate Imaging and Reporting Data System (PI-RADS), DWI is currently the dominant sequence for assessing lesions detected in the peripheral zone (PZ). Furthermore, it is a very important sequence for determining the PI-RADS category in transitional zone (TZ) lesions since the diagnostic ability of the T2W sequence is limited by the heterogeneity of stromal and glandular elements in TZ [8,9,10]. The disadvantage of DWI is its susceptibility to artifacts, and the optimal method of ADC measurement is still the subject of research [11]. Also, DWI in TZ is used in conjunction with the T2W sequence, which is the dominant sequence in TZ. Thus, the use of quantitative ADC values in TZ needs further research and validation [7].

DCE-MRI is also an important functional sequence that requires intravenous administration of a gadolinium-based contrast agent. It provides information regarding the vascularity and capillary permeability of the lesion [12]. According to currently valid PI-RADS v2.1, DCE is used in peripheral zone lesions together with DWI and T2W to determine the final score, while in transitional zone lesions it currently has no role in PI-RADS categorisation [10]. DCE-MRI also has a supporting role in complementing the examination when there are dubious imaging findings or technically suboptimal image quality as a consequence of magnetic field distortion or motion artifacts [13].

Many researchers have demonstrated that DCE-MRI has a key role in cancer localisation and in predicting cancer aggressiveness. In addition, its role is especially important in detecting residual or recurrent disease [14,15].

Although numerous studies have confirmed that DCE-MRI has great potential in the diagnosis and monitoring of prostate cancer, its role is still inadequate in the PI-RADS categorisation. What is more, there has been a notable surge in scientific discussions about abandoning the intravenous application of gadolinium-based contrast as a routine part of MRI examination of the prostate.

In recent years, the value of DCE-MRI in PI-RADS categorization has been the subject of many studies that discuss the advantages and disadvantages of its application in everyday practice [16,17,18,19,20,21,22,23]. Biparametric magnetic resonance imaging (bpMRI) consists of DWI and T2W sequences without DCE-MRI. Many studies comparing bpMRI and mpMRI concluded that bpMRI has satisfactory results in detecting clinically significant prostate cancer (csPCa) [17,18,19,20,21]. The research emphasises the shortcomings of DCE-MRI that can be avoided using bpMRI, such as extended examination duration, additional examination cost, and the potential risks of intravenous application of gadolinium-based contrast agents, like allergic reactions, renal failure, nephrogenic systemic fibrosis, and brain depositions of gadolinium.

As opposed to that, according to other research, DCE-MRI has been found to be a highly sensitive sequence in detecting csPCa and improving the accuracy of the overall examination [24,25,26,27]. That research has mainly been focused on improving the sensitivity and especially the specificity of MRI examinations of the prostate using DCE-MRI. Namely, the major strength of mpMRI of the prostate is its high negative predictive value, between 90 and 93% [28]. However, the false positive rate is too high, up to 70% according to some research, which presents a problem [29,30,31].

It is debatable whether DCE-MRI should be abandoned or whether it should be used better. Although there are many articles on the subject of bpMRI and mpMRI, they do not discuss the possible better usage of DCE-MRI. In this review, we aimed to summarise the current literature on the advantages and disadvantages of DCE-MRI, focusing on studies providing information on the potential better usability of DCE-MRI in improving the sensitivity and specificity of the prostate mpMRI examination. Furthermore, we provided a review of all categorisation systems related to the diagnosis of prostate cancer, in which the role of contrast is crucial.

## 2. Materials and Methods

This narrative review focused on DCE-MRI and its role as a diagnostic tool in detecting prostate cancer, as well as in the follow-up of patients with biopsy- or prostatectomy-proven prostate cancer. The aim of this review was to summarise the current literature regarding the clinical use of DCE-MRI as a part of multiparametric resonance imaging of the prostate. The principles of DCE-MRI data acquisition and analysis were described, along with the current role of DCE-MRI in clinical practice and its role in presently available categorisation systems. Furthermore, the advantages and disadvantages of both bpMRI and mpMRI were analysed. To conduct the literature search, various electronic databases were utilised, including Google Scholar, PubMed, and Web of Science. Also, additional studies were used from the reference list of the identified studies. The search included articles published in English until July 2023. There were no data exclusion criteria based on publication data, type of publication, or country. Articles focused on DCE-MRI, the advantages and disadvantages of intravenous contrast media as a part of mpMRI, and articles providing important background information were included.

The following keywords were used in the search: prostate cancer, clinically significant prostate cancer, biparametric magnetic resonance imaging (bpMRI), multiparametric magnetic resonance imaging (mpMRI), dynamic contrast-enhanced magnetic resonance imaging (DCE-MRI), enhanced magnetic resonance imaging, and unenhanced magnetic resonance imaging. All included images and figures are from a personal source.

## 3. Principles of DCE-MR—Acquisition and Analysis 

DCE-MRI is a T1W gradient-echo (GRE) imaging sequence with excellent temporal resolution that provides information about the enhancement pattern of tissue during the time of acquisition [32]. It also provides information on microvessel wall permeability, lesion perfusion, and the extravascular extracellular compartment. A gadolinium-based contrast agent is injected into the vascular system, and a series of images are obtained before, during, and after the contrast media has arrived in the region of interest. 

Due to its paramagnetic properties, the gadolinium-based contrast agent shortens the T1 relaxation time of the tissue in which it concentrates. The concentration of the gadolinium-based contrast agent in the region of interest reflects blood flow and leakage of contrast in extravascular extracellular space, tissue permeability, and perfusion. The malignant lesion has fast and early enhancement with early contrast wash-out compared to benign tissue due to neoangiogenesis, which is a key component of cancer growth [33,34]. As cancer grows, there is an increased need for nutrients, which is why cancers stimulate the production of vascular permeability factor and vascular endothelial growth factor (VEGF), thus stimulating the growth of new vessels that are disorganised and permeable [35].

In order to enable adequate interpretation, it is crucial to standardise DCE-MRI acquisition and analysis, which is why PI-RADS guidelines suggest imaging parameters in detail [8,9,10].

According to the latest PI-RADS v.2.1, DCE-MRI can be performed using 2D or 3D T1W GRE sequences. The latter is preferred due to its superior signal-to-noise ratio (SNR) [10]. It is also advised to use the same imaging planes for DWI sequence and for DCE-MRI with a field of view (FOV) that includes both the entire prostate and seminal vesicle. The recommended in-plane resolution is ≤2 × 2 mm with a 3 mm slice thickness and no gaps between slices [9,10]. Recommended temporal resolution is ≤15 s, and special attention must be paid to maintaining adequate spatial resolution with a time of acquisition of ≥2 min [10]. The high rate at which DCE-MRI must be obtained requires larger voxels to maintain adequate SNR. The recommended dose of gadolinium-based contrast agent is 0.1 mmol/kg (0.2 mL/kg) at an injection rate of 2 mL/s, followed by a 20 mL saline flush [8,9,10]. High time resolution and a fast injection rate are essential for detecting early arterial enhancement in prostate cancer. Concentrated IV-injected contrast media in prostate cancer tissue microvasculature and the extravascular extracellular space shorten T1 relaxation time, so that the signal intensity in the tissue of interest measured on DCE-MRI reflects both perfusion and permeability characteristics in that tissue [36]. It is recommended to use the subtraction of contrast or fat suppression technique for better contrast resolution. Subtraction of contrast has several advantages, especially if there is a post-biopsy haemorrhage or haemorrhagic cyst present. Haemorrhagic foci also show high signal intensity and can be misinterpreted, suggesting that there is early contrast enhancement. Therefore, subtraction of contrast eliminates any other T1W high signal intensity.

Another important point is the differentiation of MRI scanners according to their Tesla strength [37]. Studies comparing 1.5T MRI and 3T MRI scanners showed that 3T has better SNR, which enables increased temporal and spatial resolution [38,39,40]. Higher magnetic field strength has a longer T1 relaxation time, so the relaxation of gadolinium-based contrast agents is reduced at 3T MRI compared with 1.5T MRI. Therefore, better contrast resolution between prostate cancer and healthy tissue is achieved [41]. The use of endorectal coils is another subject of debate and research. Studies have shown that an endorectal coil improves the diagnostic quality of the overall examination by providing a higher SNR [42,43,44]. Also, studies have shown that an endorectal coil provides better accuracy in detecting residual or recurrent disease after prostatectomy or radiation therapy [37,42,45]. A significant disadvantage of an endorectal coil is discomfort for patients, as well as additional cost [46]. Further, larger studies are needed to standardise recommendations on this matter.

**Figure 2:**
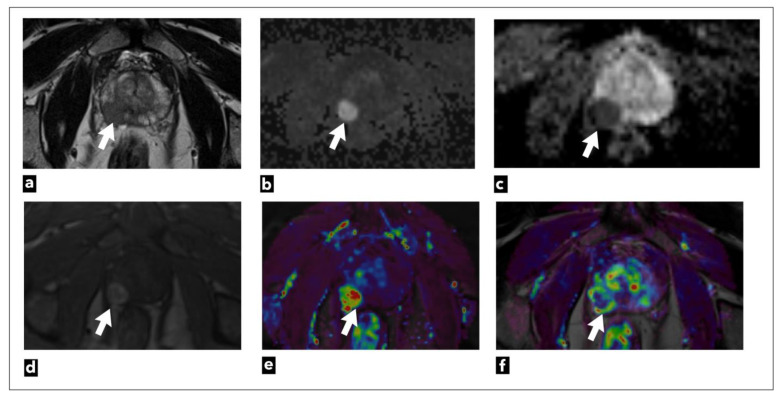
The mpMRI performed on a 75-year-old male patient shows a right peripheral zone prostate lesion (white arrow) that was confirmed to be cancer on biopsy; Gleason score 4 + 3 = 7. The images present the following: (**a**) T2W as a hypointense focal lesion; (**b**) DWI b = 1400 s/mm^3^; (**c**) ADC map demonstrates highly restricted diffusion and early contrast enhancement; (**d**) raw data; (**e**) semi-quantitative colour-coded parametric map for a wash-in; (**f**) pharmacokinetic quantitative colour-coded parametric map for Ktrans.

There are three analytic approaches to DCE-MRI analysis: qualitative, semi-quantitative, or quantitative [47]. Also, it can be analysed from raw data or using coloured parametric maps (Figure 2).

The currently valid PI-RADS v2.1 categorisation includes qualitative visual analysis, the simplest method of analysis, since it does not require special software, but can be analysed using a simple Picture Archiving and Communication System (PACS) workstation. This method relies only on the visual assessment of early arterial contrast accumulation in lesions previously observed as suspicious on T2W and DWI sequences. If early contrast accumulation is observed, the DCE-MRI finding is considered positive. However, DCE-MRI is considered negative [13] if early contrast accumulation is not observed or if diffuse contrast accumulation that does not correspond to the location of the T2W/DWI suspicious lesion is observed. The disadvantage of this method is the lack of standardisation and objectivity since interpretation is based on subjective assessment [47].

**Figure 3:**
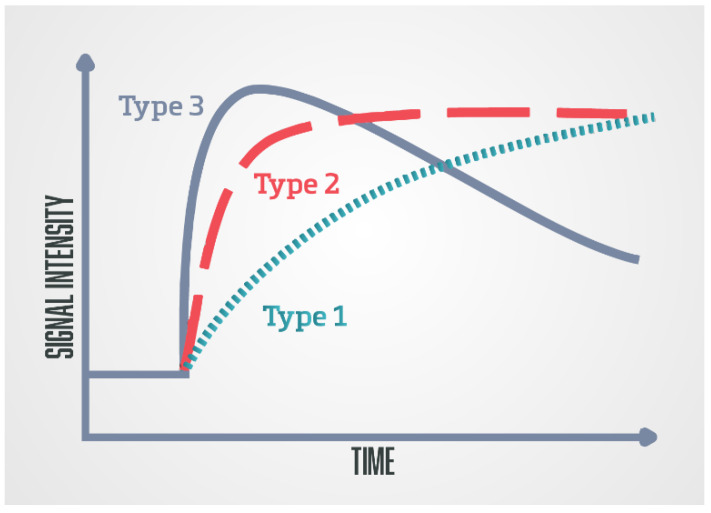
Schematic diagram of the DCE-MRI time-signal intensity (semi-quantitative analysis)/time-concentration (quantitative analysis) enhancement kinetic curves: Type 1 (progressive), type 2 (plateau), and type 3 (wash-in and wash-out).

The second analytic method is a semi-quantitative method that analyses the kinetics of contrast accumulation on the observed lesion, wash-in, i.e., the arrival of the contrast agent in the lesion, wash-out, time to peak (TTP), and peak enhancement (PE) [48]. Using special software, the signal-intensity time curves are created [47]. There are three types of curves: type 1 curve (progressive) is a persistent curve in which there is a gradual increase in contrast accumulation and it is characteristic of benign changes; type 2 curve (plateau) is a curve in which there is an early and sudden contrast accumulation followed by a plateau; it can be seen in both benign and malignant focal lesions; and type 3 curve (wash-in and wash-out), a curve where there is an early and sudden accumulation of contrast followed by wash-out of the contrast agent, and this type of curve indicates the presence of prostate cancer (Figure 3) [49].

Using kinetic curves is a visually simple and easy-to-use method of analysis, but the shape of the curve highly depends on the injection quality, which makes it less reproducible [37]. Also, motion artifacts that result from peristalsis or patient motion artifacts can result in misregistration between successive slices, which is seen as noise in the curves. Special software used for image post-processing enables automatic correction of motion artifacts [13]. Parameters obtained with this method can be displayed and analyzed as parametric coloured maps which are fused with the T2W sequence for easier anatomical orientation [50].

The disadvantage of this method is that the values are estimated only based on the change in signal intensity in the observed lesion, without a physiological or empirical model, and the change in signal intensity in the tissue depends on the parameters that are not taken into account with this method like sequence parameters, the dose and type of injected contrast agent and on the characteristics of the tissue itself [48].

Also, this method has a weak possibility of distinguishing between benign and malignant lesions since all three curves can be seen in both benign and malignant lesions, and according to some research, there is a lack of reproducibility and accuracy of this method [51]. Mainly due to these reasons, the semiquantitative method, which was initially included in PI-RADS v.1 was removed from PI-RADS v.2.(8-10).

The third analytic method is the quantitative method, which is the most sophisticated DCE method. It is based on the measurement of pharmacokinetic parameters using one of the pharmacokinetic models [49]. The most commonly used Tofts model [52].

The parameters obtained using this method are K_trans_ (constant of transendothelial transfer of contrast medium from intravascular space to extravascular extracellular space), K_ep_ (constant of transendothelial transfer of contrast from extravascular extracellular space back to intravascular space), V_p_ (plasma volume in relation to total volume tissue), and V_e_ (tumour extravascular extracellular space volume) [2,53]. These parameters provide information about microvascular permeability in observed tissue [54]. The obtained parameter values can also be displayed and analysed as parametric coloured maps fused with the T2W sequence for easier anatomical orientation. Time-concentration curves can be generated with this method using special software.

The signal intensity of some tissue seen on DCE-MRI is not proportional to contrast concentration in that tissue, and therefore pharmacokinetic models take into account the amount and concentration of contrast as well as the time of contrast arrival in the supplying blood vessel (arterial input function; AIF) [2,48,52,55]. AIF can be determined individually, or population-based AIF can be used [56]. Individual AIF is more patient-specific because every patient has a different haemodynamic characteristic (heart rate, blood volume), but it is considered valid to use population-based AIF in clinical use since it is time-saving, and less operator-dependent [57].

Research analysing parameters in this method has shown that there are increased values of K_trans_ and K_ep_ in prostate cancer compared to normal prostate tissue [14].

This method also requires special software, which makes it currently less available in everyday clinical practice. Furthermore, it prolongs the time required for the analysis of the obtained magnetic resonance images and may also give abnormal findings in cases of some benign changes, such as nodules in BPH, or prostatitis [58]. This method is also influenced by motion artifacts since they can make noise in curves and lead to several limitations in curve fitting with the pharmacokinetic model. However, software can automatically reposition the successive slices so that they align better [13].

For these reasons, this method was not included in PI-RADS v.1 nor in PI-RADS v.2, but research dealing with this method shows the advantages of this method and the possibility of its implementation in everyday clinical practice. In magnetic resonance imaging of other organic systems, this method has been proven effective and applicable [59,60,61,62].

## 4. Current Role of DCE-MRI in Clinical Practice

Magnetic resonance imaging has played a significant role in the diagnosis and staging of patients with prostate cancer since 1982. Back then, a device with a magnetic strength of 0.08 Tesla was used [63]. At that time, the purpose of the examination was only to evaluate the morphological characteristics of the tissue. T1 pulse turbo spin echo sequence (T1 TSE) and T2 pulse turbo spin echo sequence (T2 TSE) were used for locoregional staging of the disease in patients with previously biopsy-proven prostate cancer. MRI imaging had limited value in distinguishing benign changes from clinically significant cancer and non-clinically significant cancer [64].

The development of technology has brought improvements to magnetic resonance imaging by manufacturing increasingly powerful devices with new functional sequences, such as dynamic contrast enhancement (DCE), diffusion-weighted imaging (DWI), and magnetic resonance spectroscopic imaging (MRSI). This has led to the development of a multiparametric examination of the prostate based on a combination of the mentioned techniques [8]. In addition to providing information on the morphology of the prostate and surrounding tissue using functional sequences, mpMRI also provides functional information on the metabolic activity of the tumour using spectroscopy, information on tumour angiogenesis using a dynamic contrast-enhanced study, and information on tumour cellularity using the diffusion technique. There was a lack of standardisation in performing mpMRI and interpreting the findings, and therefore the AdMeTech Foundation established an international group for prostate MRI in 2007. It included world-class experts from the European Society of Urogenital Radiology (ESOR) and the American College of Radiology (ACR), whose task was standardisation of the recording protocols and radiological reports [65]. This is how PI-RADS (Prostate Imaging Reporting and Data System) v.1 was created in 2012, followed by PI-RADS v.2 in 2016, and an updated version, PI-RADS v.2.1, in 2019 [8,9,10].

Prostate mpMRI enables precise localisation of focal changes in the prostate gland, their characterisation, and the assessment of clinical significance. This results in a more precise biopsy and reduces the unintended consequences of invasive diagnostic methods, excessive and redundant therapy, and their unjustified use, while at the same time enabling advanced detection of prostate cancer [8].

According to recent research, the negative predictive value of multiparametric examination of the prostate with magnetic resonance is 90–92%, and the positive predictive value is 30–52% [66,67].

The accuracy of mpMRI is mainly questioned due to its high false-positive rate [29,67]. There are numerous studies dealing with increasing the specificity of mpMRI by choosing the most appropriate feature for distinguishing prostate cancer from specific benign lesions that are the cause of false positive findings, such as atrophy, inflammation, benign prostatic hyperplasia (BHP), and prostatic intraepithelial neoplasia (PIN) [68,69,70,71,72,73,74,75,76]. According to those studies, DCE has potential for distinguishing some benign changes from prostate cancer and increasing the specificity and sensitivity of the overall examination.

DCE-MRI provides information on the vascularisation of observed changes and the permeability of blood vessels [47]. Neoangiogenesis is a key component of cancer growth [77]. As cancer grows, the need for nutrients increases, which is why more aggressive tumours stimulate the production of vascular permeability factor (VPF) and vascular endothelial growth factor (VEGF). Thus, the growth of new vessels is stimulated, but these vessels are disorganised and permeable, unlike vessels in healthy tissue [78]. This is shown in the DCE sequence by rapid early arterial contrast accumulation in the focal change and rapid contrast wash-out [79].

Numerous studies have dealt with the sensitivity and specificity of the DCE sequence in the detection of prostate cancer, and the results range from 74–96% in specificity and 46–96% in sensitivity, depending on the imaging technique, tumour size, and diagnostic criteria [80,81,82,83,84].

**Figure 4:**
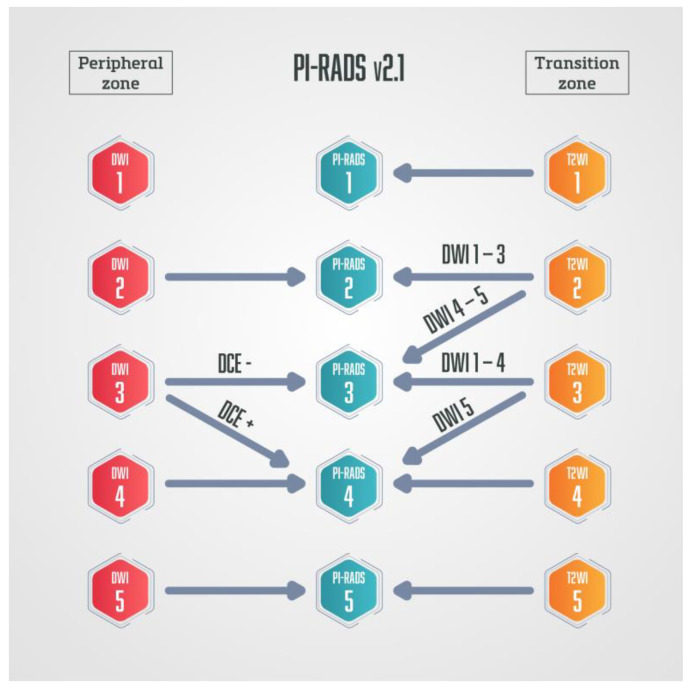
Schematic diagram of PI-RADS v.2.1. in which DCE-MRI has a role only in peripheral zone lesions categorised on DWI as PI-RADS 3—if those lesions show early arterial accumulation of contrast, DCE-MRI is considered positive, and lesions are upgraded to PI-RADS 4.

The semi-quantitative method was used in PI-RADS v.1 [8], while the qualitative method of DCE analysis is used in currently valid PI-RADS v.2.1 [9,10]. In the PI-RADS categorisation DCE-MRI has a clear application only in peripheral zone lesions characterised on DWI as PI-RADS 3. If there is an early arterial contrast accumulation in the observed change, it is upgraded to the PI-RADS 4 category, and if there is no early contrast enhancement, the PI-RADS 3 category remains (Figure 4) [9,10].

According to PI-RADS v.2, DCE sequence has no role regarding focal changes in the transition zone, but it is still recommended to record the DCE findings in the transition zone [9,10]. Although the PI-RADS scoring system is widely accepted, there is also an alternative Likert score [85]. The Likert scale has been recommended by National Institute for Health and Care Excellence (NICE) guidelines in the United Kingdom [86]. On the Likert scale, clinical parameters, genetics, and PSA density are taken into account. All sequences are considered equally important, so there is no dominant sequence [87]. Unlike the PI-RADS score, where DCE-MRI is used only as a secondary sequence in peripheral zone cancer, in the Likert score, DCE can have the role of primary sequence for both transitional and peripheral zone cancer [86,87]. Unlike PI-RADS, where DCE can only upgrade the score, Likert DCE can both upgrade and downgrade the score. Also, DCE-MRI is considered positive in PI-RADS only if there is a focal enhancement in a suspicious lesion, while in Likert diffuse early enhancement can also be considered a positive DCE-MRI finding [87,88]. Therefore, DCE-MRI has a more significant role in the Likert score than in PI-RADS.

The role of DCE-MRI is quite different in these two scoring systems. Its role in the PI-RADS categorisation is of secondary importance, and thus it is the subject of debate whether it should be a standard part of the examination [16,17,18,19,20,21,22,23]. Although DCE cannot be used alone, due to its high spatial resolution when compared to DWI and also due to its high contrast resolution, it can significantly help in the detection of prostate cancer in cases where TW and DWI sequences are of limited diagnostic value due to artifacts or in cases of prostate cancer located in some challenging locations, like subcapsular or apical locations [37].

Another scoring system, in which DCE plays a much more important role, is called Prostate Imaging for Recurrence Reporting (PI-RR). It was suggested by experts from the European Society of Urologic Imaging, the European Society of Urogenital Radiology, and members of the Prostate Imaging-Reporting and Data System (PI-RADS) Steering Committee in 2021 [89].

**Figure 5:**
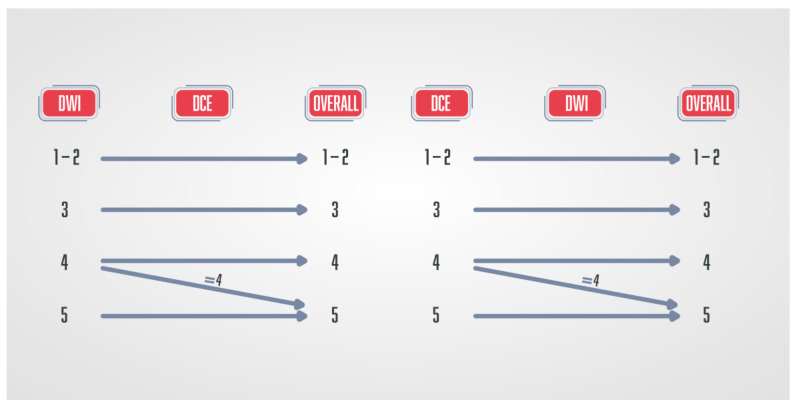
Schematic diagram of the PI-RR score in patients after radiation therapy. DWI and DCE have the key role, and the final score is defined by the sequence with a higher score.

**Figure 6:**
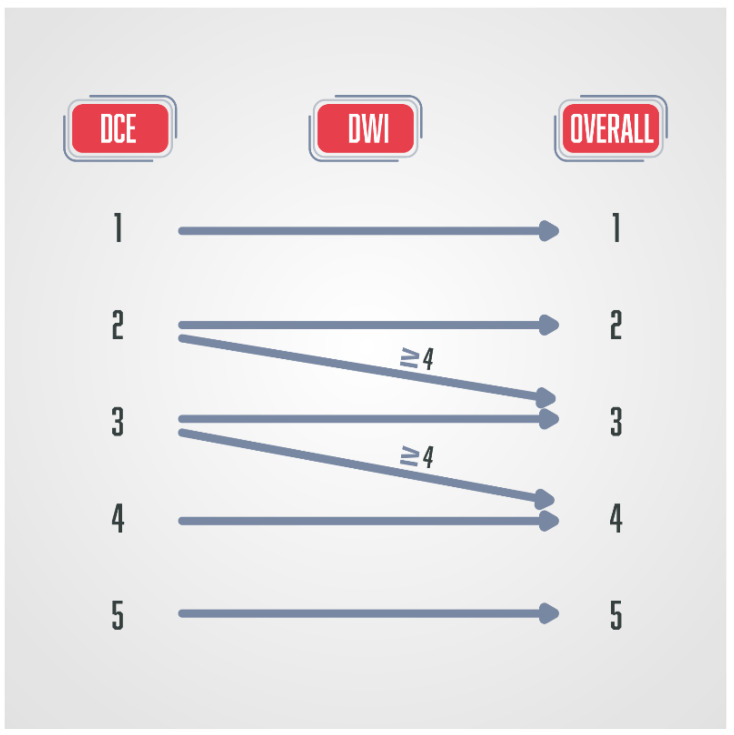
Schematic diagram of the PI-RR score in patients after radical prostatectomy, where DCE has a key role in the final score.

PI-RR is a scoring system that estimates the probability of prostate cancer local recurrence following radiation therapy (RT) (Figure 5) or radical prostatectomy (RP) (Figure 6).

The most common location of prostate cancer recurrence after radiation therapy is the primary prostate cancer location [90]. Radiation therapy can result in dramatic changes in the prostate gland morphology, anatomy, and signal intensity seen on T2W sequences due to fibrosis, inflammation, or atrophy [91]. For those reasons, T2W sequences are used only for the anatomical orientation and precise location of detected suspicious lesions in detecting prostate cancer recurrence after radiation therapy, but do not play a part in deciding the final score [89]. DWI and DCE sequences have the key role in determining the final PI-RR score, and the sequence that has a higher score determines the final score [89,92]. DCE-MRI shows recurrent prostate cancer as a hypervascular lesion due to the formation of new vessels with increased vascular permeability [93].

The drawback of DCE-MRI is the fact that if it is performed within the three months following RT, radiation-induced inflammatory changes result in falsely positive hypervascular areas [89,94].

DCE-MRI plays a crucial role as a dominant sequence in detecting prostate cancer recurrence after radical prostatectomy [89,92]. Prostate cancer recurrence is characterised by early arterial enhancement with an early wash-out that is easily visible on DCE-MRI, while there is slow or no enhancement in postoperative cicatricial and granulation tissue [95]. In postoperative PI-RR scoring, T2W also has a role only in anatomical and morphological assessment, and DWI has the secondary role in upgrading the score PI-RR 2 to PI-RR 3 and PI-RR 3 to PI-RR 4 when the DWI score is ≥4 [89,92]. Also, DCE is of limited value in the first three months after a radical prostatectomy [89], as well as in post-radiation imaging assessment, which are its major disadvantages. Studies have observed the benefit and superiority of DCE-MRI compared to T2W sequence in the detection of prostate cancer residue or recurrence [96,97]. Haidar et al. observed that the dynamic contrast study has a sensitivity of up to 72%, a positive predictive value of up to 46%, and a negative predictive value of up to 95%. In comparison, the T2 sequence has a sensitivity of up to 38% when detecting recurrence/residue after radiotherapy in the peripheral zone, a positive predictive value of up to 24%, and a negative predictive value of up to 88% [96].

Another important clinical role of DCE is in the tumour, node and metastasis (TNM) classification.

DCE, together with the T2W sequence, helps to distinguish between the T2 and T3 stages; that is, it facilitates the assessment of the extracapsular spread of prostate cancer, which is characteristically associated with a worse prognosis [98].

Studies have shown changes in pharmacokinetic parameter values in patients undergoing chemotherapy or radiotherapy as a response to applied therapy [99,100].

The results of these studies are useful since they suggest that, in addition to monitoring therapeutic responses in patients, prostate mpMRI can also be used to detect patients who are resistant to androgen deprivation therapy.

## 5. Biparametric or Multipametric MRI—Future Perspective

Although mpMRI of the prostate is a widely accepted method in early prostate cancer diagnosis, there are more and more studies comparing it with bpMRI, considering that mpMRI includes DCE-MRI and bpMRI does not include DCE-MRI sequence. Scoring in bpMRI is based only on the multiplanar T2W sequence and DWI. Using contrast in prostate MRI is controversial for many reasons. Firstly, DCE-MRI can prolong the examination by up to 45 min, resulting in more motion artifacts since it can be very uncomfortable for patients, especially when an endorectal coil is used or when the patients suffer from claustrophobia [46]. Secondly, using intravenous contrast media increases the cost of the examination. There is also a health risk associated with the use of gadolinium-based contrast agents, such as deposition of gadolinium in the central nervous system, allergic reactions, and nephrogenic systemic sclerosis [16]. For these reasons, gadolinium-based contrast media should be used as a part of the examination only when necessary. It is therefore not surprising that numerous studies have focused on questioning the role of DCE in prostate MRI.

Many studies comparing the diagnostic performance of bpMRI and mpMRI found they had similar sensitivity and specificity in detecting clinically significant prostate cancer, which implies that routine intravenous application of gadolinium-based contrast media does not significantly improve the diagnostic value of the examination [17,101,102,103,104,105,106,107,108,109,110,111,112,113,114].

These studies confirm that DCE-MRI has been proven useful in cases of suboptimal diagnostic quality of DWI or T2W, but in PI-RADS v.2.1 categorisation. DCE-MRI has a supplementary role only in peripheral zone lesions categorised as PI-RADS 3, where a positive DCE-MRI upgrades the lesion to category PI-RADS 4. Considering the risk and disadvantages of intravenous use of gadolinium-based contrast agents with minimal diagnostic benefit, these researchers question the need to include DCE-MRI in the standard protocol.

In contrast to these, other studies have found that including DCE-MRI in the examination can significantly increase the accuracy of clinically significant prostate cancer detection in peripheral zone lesions [24,115,116]. The research by Greer et al. indicates that early arterial accumulation of contrast in DCE-MRI increases the likelihood of detecting clinically significant cancer, even in the PI-RADS 2 category [115]. Regarding the transitional zone (TZ) lesions, where DCE-MRI currently plays no role according to the PI-RADS categorisation, the research by Rosenkratz et al. suggested revalidation of the PI-RADS categorisation and DCE role in transitional zone (TZ) lesions [117]. They found that when the morphological characteristic of unencapsulated sheet-like enhancement in TZ lesions is included in upgrading PI-RADS categories 3 to 4, 33.3–57.1% of the upgraded lesions were proven to be clinically significant cancers.

According to some studies, bpMRI results in more false-positive findings than mpMRI since it lacks a DCE sequence [112,118,119]. In contrast, according to other studies, mpMRI has more false positive findings due to some benign changes that can overlap prostate carcinoma characteristics on DCE, especially in TZ lesions [98,120,121]. Although the conclusion the researchers in both cases came to states that too many false positives are a better option than missing any clinically significant prostate cancer, too many false positive findings result in unnecessary biopsies with all the potential risks and possible complications for patients, as well as unnecessary expenses. The psychological component of anxiety patients experience due to the diagnosis of possible prostate cancer should not be ignored either [122]. It has been evident that some of the most common causes of false-positive MRI findings are benign changes, like atrophy, inflammation, benign prostatic hyperplasia (BHP), and prostatic intraepithelial neoplasia (PIN) [69,70,71,118]. Moreover, some normal anatomical structures can be mistaken for prostate cancer [118,123]. Although experience and education are invaluable in interpreting the findings, it is still not always possible to distinguish benign from malignant changes with absolute certainty. Therefore, numerous studies are focused on finding a non-invasive method of distinguishing prostate cancer from benign causes of false positive MRI findings [68,72,73,74,118,124,125,126,127].

Significant research on the subject is that of Litjens and colleagues, who investigated which characteristics are the best for distinguishing certain benign changes of the prostate from prostate cancer [68] among currently available morphological and functional sequences in mpMRI examination of the prostate. The benign changes they investigated were PIN, inflammation, atrophy, and benign prostatic hyperplasia. They concluded that the ADC map is the most significant sequence of multiparametric prostate magnetic resonance examination for distinguishing PIN from prostate cancer, followed by the Gauss (XX, s = 4.1) T2 sequence and Hess (K_trans_). Hess (V_e_) was the most significant for prostate atrophy, followed by Hess (b = 800 s/mm^2^) and K_trans_. Hess (T2 map) was the most useful for inflammation, followed by ADC and Hess (Ve), while Hess (b = 800 s/mm^2^) was the most important for benign prostatic hyperplasia (BHP), followed by Hess (T2 map) and ADC map [68].

They concluded that using the most specific characteristics of mpMRI examination for each individual benign change increases the accuracy in differentiating prostate cancer from benign changes, especially PIN and atrophy. This means that parameters obtained by DCE-MRI can also play an important role in increasing the specificity of the examination.

Another study calculated the tissue-to-muscle ratio of quantitative DCE parameters obtained from prostate cancer, normal prostate tissue, and obturator internus muscle. It introduced a model that combines K_trans_ and iAUC as two parameters with the best individual predictive value for prostate cancer detection, with a proposed cut-off value that has 100% sensitivity and 64.28% specificity [76]. That research suggests it is possible to make better use of a DCE sequence than is currently used or use it in a different manner, with a resultant increase in both the sensitivity and specificity of the examination. These studies are not ready for clinical application, but strongly suggest the need for prospective studies on significantly larger samples of patients.

According to some other studies, up to 80% of DCE sequences performed as a part of the mpMRI examination do not affect the final PI-RADS score [119,128]. On the contrary, research by Taghipour et al., which focused only on peripheral zone indeterminate lesions categorised as PI-RADS 3, found that DCE-MRI altered the final score to PI-RADS 4 in 21.5% of lesions and was accurate in 68.9% of indeterminate lesions [129].

Another significant study by Stanzione and colleagues showed that it is possible to assess extraprostatic extension without intravenous administration of a contrast medium using data extracted from bpMRI [130]. They combined texture analysis (TA) and machine learning (ML) methods on data extracted from the T2W sequence and ADC map in patients who underwent radical prostatectomy and concluded that it is possible to predict extraprostatic extension using TA and ML.

Encouraged by research showing that bpMRI has diagnostic accuracy comparable to mpMRI, some studies suggest using an even shorter examination protocol called dual-pulse MRI (dpMRI), which consists of an axial T2W sequence and DWI [109,131,132]. These studies proved that dpMRI also has diagnostic accuracy in detecting clinically significant prostate cancer compared to mpMRI. But in another study, Stanzione and colleagues compared the diagnostic performance of dpMRI, bpMRI, and mpMRI in detecting extraprostatic extension of prostate carcinoma and concluded that bpMRI and mpMRI have similar accuracy, while dpMRI had a much worse correlation with histopathology findings [132].

There are more and more studies in favour of bpMRI in everyday clinical practice, since it saves time and money and reduces the health risk for patients caused by the use of gadolinium-based contrast agents. Therefore, the PI-RADS Steering Committee has published a paper in which they express and explain their point of view, declaring that the benefits of initial bpMRI examinations “need to be carefully weighed against the effects on radiologic image assessments and diagnostic performance” [133].

**Figure 7:**
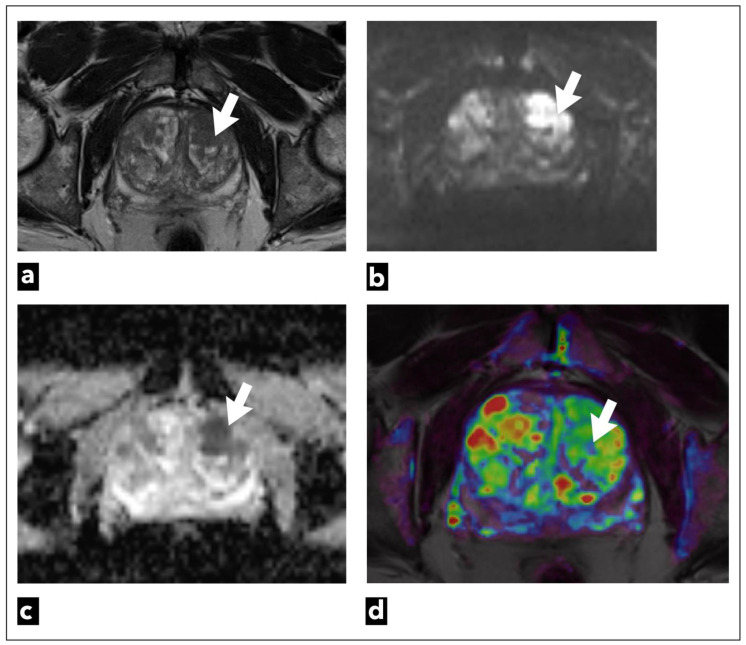
mpMRI shows a left transitional zone prostate lesion (white arrow) in a 69-year-old male patient, which was confirmed to be cancer on biopsy; Gleason score 4 + 3 = 7. It can be seen on (**a**) T2W as a hypointense focal lesion. The lesion is obscured on (**b**) DWI b = 1400 s/mm^3^ and (**c**) ADC maps that are of suboptimal diagnostic quality due to artifacts. DCE-MRI does not demonstrate early contrast enhancement on the (**d**) pharmacokinetic quantitative colour-coded parametric map for K_trans_.

**Figure 8:**
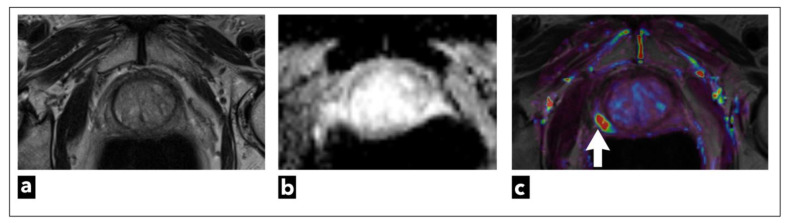
mpMRI shows a right peripheral zone prostate lesion (white arrow) in a 72-year-old male patient, which was confirmed to be cancer on biopsy; Gleason score 3 + 3 = 6. The images present (**a**) T2W as a lightly hypointense focal lesion, obscured on (**b**) ADC map due to artifacts, and the most noticeable on (**c**) DCE-MRI, which demonstrates early contrast enhancement on the pharmacokinetic quantitative colour-coded parametric map for K_trans_.

They suggest dividing patients into three categories: low, intermediate, and high risk, and using DCE-MRI in intermediate and high-risk patients. However, when it comes to low-risk group patients, such as patients undergoing screening where there is a higher risk of overdiagnosing, the committee regards bpMRI as a potentially reasonable method [133]. Examinations categorised as PI-RADS 1 or 2 can be scored using only T2W sequence and DWI, which can also be used in the majority of highly suspicious examinations, especially those categorised as PI-RADS 5 (Figure 7). DCE-MRI can have a crucial role when it comes to small cancers, cancers that are found in locations that may be more difficult to analyse with T2W or DWI sequences, like the apex of the prostate, the subcapsular area of both the peripheral and central zones, or when T2W and DWI are of suboptimal diagnostic quality (Figure 8) [134].

Also, using DCE-MRI has been proven very useful to less experienced radiologists, helping them to find less obvious prostate cancers with more confidence [135]. When it comes to more experienced readers, studies have shown an increment in the number of indeterminate cases in bpMRI examinations, both for PI-RADS and Likert categorisations [119,136]. Implementing bpMRI in everyday clinical practice would require some adjustments and strict guidelines on when to use contrast. It is essential to have high-quality T2W and DWI sequences, reading radiologists with sufficient experience, and validate biopsy decisions in correlation with clinical risk for prostate cancer. In indeterminate cases or cases with suboptimal quality of T2W and DWI sequences, patients should be recalled for additional DCE-MRI sequences [133]. Recalling patients can result in a new problem of delayed diagnosis and can be a complication for some patients.

Some research suggests that calculating PSA density from T2W images and PSA value could help elect high-risk patients in whom gadolinium-based contrast medium should be administered [137].

Although many studies suggest that DCE-MRI should not be part of the routine examination, based on the currently available literature, the DCE-MRI sequence should not be completely abandoned.

Several studies that have proven similar diagnostic value in bpMRI and mpMRI have excluded dubious cases where small cancers are difficult to detect due to their location or the diagnostically suboptimal quality of T2W and DWI sequences [119].

According to currently available literature, the potential role of pharmacokinetic parameters in increasing the sensitivity and specificity of the mpMRI examination derived from quantitative DCE-MRI analysis is very promising and warrants further research [34,76,80,122,138,139,140,141,142,143,144].

Radiogenomics, a branch of radiology that is a promising part of the future perspective, should also be mentioned here. In the time of rapid development of technology and artificial intelligence, radiogenomics enables the connection of imaging characteristics with their genomic characteristics, mainly due to its multidisciplinary approach, which facilitates a personalised approach to patients [145].

When it comes to prostate cancer, genetic biomarkers such as tumour suppressor gene phosphatase and tensin homolog (PTEN) have been proven to have an important role in prostate carcinogenesis and have great prognostic value regarding advanced metastatic disease, mortality, and the potential recurrence of the disease [146]. It is especially valuable in lower-risk cancers, such as Gleason score 6, where PTEN deletion suggests the existence of higher-grade or already advanced disease not detected by prostate biopsy [147]. The research by McCann et al. found a negative correlation between quantitative pharmacokinetic parameters derived from DCE-MRI, K_ep_, and PTEN expression [148]. Also, several studies have so far shown that some quantitative pharmacokinetic parameters, especially K_trans_, are positively correlated with the Gleason score [139,149,150,151].

## 6. Conclusions

Since the European Association of Urology Prostate Cancer guidelines have advised performing mpMRI examinations of the prostate before prostate biopsy, there is increased clinical demand for MRI examinations [152]. Shortening the duration of the examination by eliminating the DCE-MRI sequence would allow an increase in the number of examinations, reduce the health risk associated with the application of gadolinium-based contrast media, and reduce the cost of the examination. Another problem in clinical practice is the high false-positive rate of mpMRI findings that increases demand for biopsy, which brings potential health risks for patients undergoing this invasive procedure and also constitutes a financial burden on the health system. There is more and more research that suggests DCE-MRI is not a necessary part of the examination, as well as research that suggests DCE-MRI can increase the sensitivity and specificity of the examination and has underutilised potential. There is a lack of consensus between researchers who support bpMRI and those who believe that DCE-MRI should not be eliminated from the standard examination. Recently, there has been more and more research showing that DCE-MRI and bpMRI have greater potential in cancer diagnosis, especially using machine learning and radiogenomics. Although these studies have promising results, they are not clinically applicable, and most of them emphasise that additional multicentre studies are needed on larger patient samples in order to validate the results and incorporate the methods into daily clinical practice.

There is a need to update currently used systems in order to increase the sensitivity and specificity of MRI examination of the prostate and also to satisfy the growing clinical demand for MRI examination while preserving the quality and accuracy of the examination. Further studies, including a larger group of patients, are required to validate the value of the DCE sequence in mpMRI examination and find a way to better and more fully utilise the DCE sequence, not only in the peripheral zone but also in transitional zone lesions. 

Also, it is necessary to establish precise clinical guidelines on when to use intravenous gadolinium-based contrast media, along with risk and benefit assessments.

## Data Availability

No new data were created or analyzed in this study. Data sharing is not applicable to this article.
